# Supplementary figures and images for: Genomic sequencing and neutralizing serological profiles during acute dengue infection: A 2017 cohort study in Nepal
Source: PLOS Glob Public Health. 2024 Nov 13;4(11):e0002966. doi: 10.1371/journal.pgph.0002966 (PMC11560038; doi:10.1371/journal.pgph.0002966)

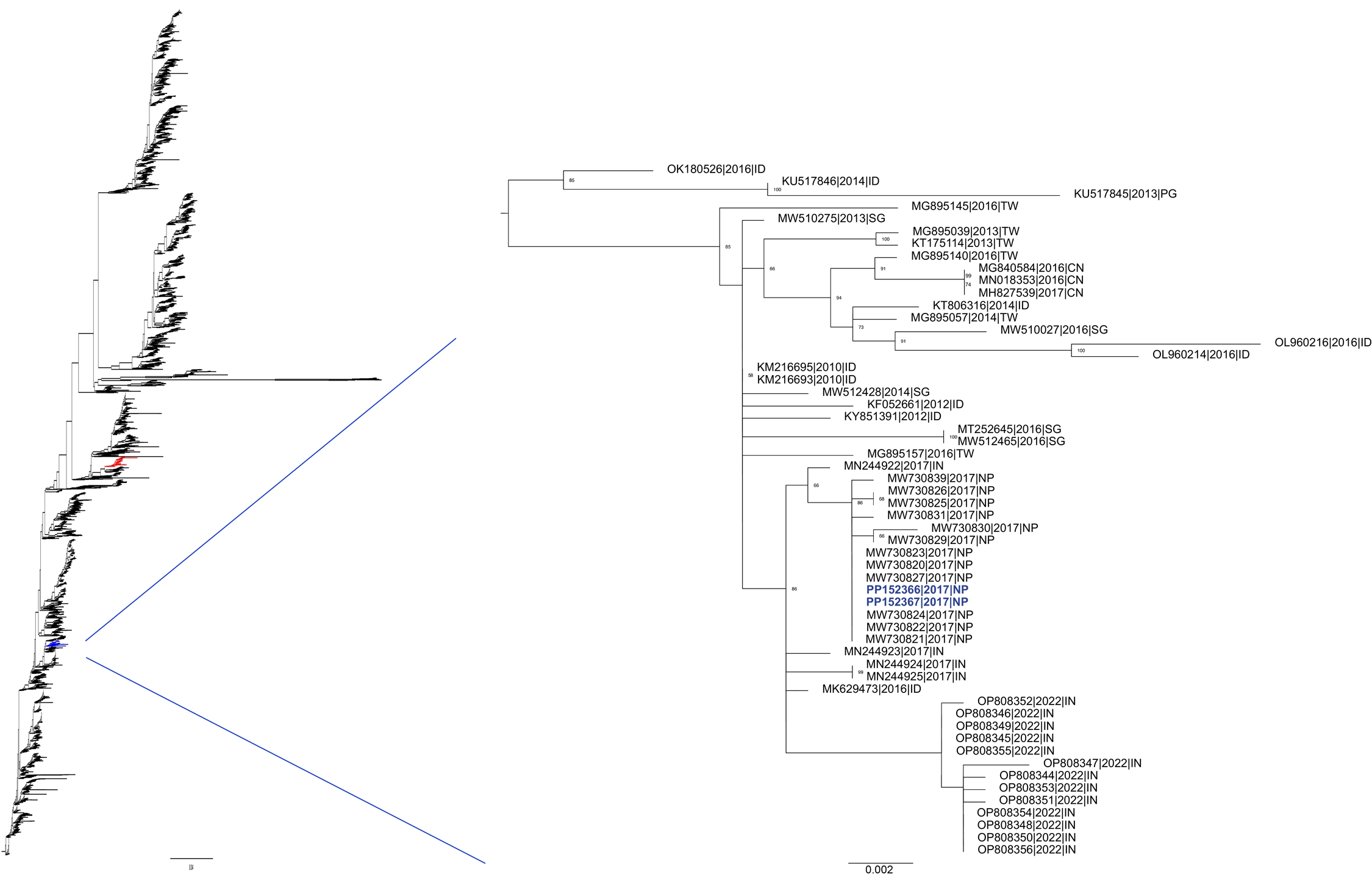

Supplement: S1 Fig — Phylogenetic tree comparing E gene sequences of the two 2017 DENV2 strains from Nepal in the current study (blue font, PP152366|2017|NP and PP152367|2017|NP) with other E gene sequences in NCBI. Strains are labeled by GenBank ID, followed by the year and country of isolation (CN, China; ID, Indonesia; IN, India; NP, Nepal; PG, Papua New Guinea; SG, Singapore; TW, Taiwan). (TIF) [file pgph.0002966.s001.tif]
